# Evaluation of the rate of residual polystomatic sublingual acinar tissue without tunneling dorsal to the digastricus using a ventral or ventrolateral approach in cats

**DOI:** 10.1111/vsu.70067

**Published:** 2025-12-15

**Authors:** Ronan A. Mullins, Irene Marirrodriga Larrocha, Cristina Ortega Jusdado, Ignacio Otero Balda, Pamela A. Kelly

**Affiliations:** ^1^ Department of Small Animal Surgery, Section of Small Animal Clinical Studies University College Dublin Dublin Ireland; ^2^ Department of Veterinary Pathobiology School of Veterinary Medicine Dublin Ireland

## Abstract

**Objective:**

To evaluate the rate of residual polystomatic sublingual acinar tissue if tunneling dorsal to the digastricus muscle and dissection to the sublingual caruncle is not performed during mandibular‐sublingual sialadenectomy in cats.

**Study design:**

Cadaveric study.

**Sample population:**

A total of 10 feline cadavers.

**Methods:**

Mandibular‐sublingual sialadenectomy was performed bilaterally in 10 feline cadavers using a ventral or ventrolateral approach in a randomized fashion. Salivary ducts were dissected as far rostral as possible with retraction of the masseter and digastricus muscles until they traveled dorsal to the digastricus muscle, where a ligature was applied. The position of the lingual nerve was identified. Following tunneling, dissection continued rostrally to the sublingual oral mucosa, where ducts were excised. Histology of dissected tissue rostral to the ligature was performed.

**Results:**

Histology identified evidence of salivary acinar tissue in 13/19 (68.4%) specimens ‐ 7/10 (70.0%) of specimens obtained with the ventral approach and 6/9 (66.7%) of those obtained with the ventrolateral approach (*p* > .99). In all cases, the lingual nerve was identified with retraction of the masseter and digastricus muscles without tunneling.

**Conclusion:**

Tunneling dorsal to the digastricus muscle exposed additional polystomatic salivary acinar tissue in most cases.

**Clinical significance:**

Tunneling dorsal to the digastricus via a ventral or ventrolateral approach may decrease the rate of polystomatic sublingual acinar tissue being left in situ in clinical cases.

## 1 INTRODUCTION

Salivary mucoceles or sialoceles are characterized by extravasation and accumulation of saliva in the tissues surrounding a salivary gland or duct and are uncommonly diagnosed in cats.^1^, ^2^, ^3^, ^4^, ^5^, ^6^, ^7^, ^8^, ^9^, ^10^, ^11^, ^12^ Two of the largest retrospective studies to date,^1^, ^2^ which recruited cases from 17 veterinary hospitals over a 12–15‐year study period, include a total of 40 cases. In comparison to dogs, cats have a total of five major salivary glands, including the mandibular, sublingual, zygomatic, parotid and molar, with the latter not present in the dog.^8^, ^11^ In most cases, the cause is unknown (idiopathic) but they can occur secondary to trauma (including iatrogenic), foreign bodies, sialoliths, and neoplasia.^1^, ^2^, ^3^, ^5^, ^11^, ^13^

Sialoceles can present in a variety of locations in cats, including sublingual (ranula), cervical, facial, zygomatic, and pharyngeal, with sublingual sialoceles most common, however, one recent study found an equal occurrence of sublingual and cervical presentations in this species.^1^, ^5^, ^6^ Clinical signs can include ptyalism/hypersialorrhoea, dysphagia, a fluctuant non‐painful swelling, respiratory distress, stertor/stridor, exophthalmos, intermittent vomiting, and lethargy.^2^, ^3^, ^5^, ^6^, ^11^, ^12^ The mandibular‐sublingual salivary gland‐duct complex (MS‐GDCx) is most commonly involved in cats and can result in the formation of sublingual, cervical or pharyngeal sialoceles.^3^, ^5^, ^6^, ^7^, ^12^, ^14^

The mandibular and sublingual salivary glands are juxtaposed in cats but not as intimately associated as in dogs.^11^, ^14^ Both glands empty by means of separate ducts at the sublingual caruncle, just lateral to the lingual frenulum.^11^, ^14^ The mandibular duct exits the gland medially and runs rostromedially in close association with the sublingual gland. The sublingual gland extends rostrally as the sublingual duct, running in close association with the mandibular duct medial and dorsal to the digastricus muscle.^11^, ^15^ Before exiting at the sublingual caruncle, salivary ducts cross dorsal to the lingual nerve, a branch of the trigeminal nerve.

Treatment options for sialoceles include drainage, marsupialization, and sialadenectomy, with surgical excision of the affected salivary gland recommended in most cases.^1^, ^2^ In the case of mandibular‐sublingual sialadenectomy, it is recommended that salivary ducts be dissected as far rostral as possible in order to remove all salivary glandular tissue, particularly if a ranula is present.^9^ Both ventral and (dorso)lateral approaches for removal of mandibular and sublingual salivary glands have been described in cats.^1^, ^2^, ^5^, ^6^ With both approaches, tunneling of the MS‐GDCx dorsal to the digastricus muscle has been described.^1^ This technique has been found to increase the length of salivary duct exposure and the completeness of excision in canine cadavers.^16^ However, on the basis of their smaller size, the less robust nature of the feline digastricus muscle, and possible differences between species in the distribution of glandular tissue along the length of the mandibular and sublingual ducts, the value of performing this technique in cats is unknown.

The objective of this study was to evaluate the rate of residual polystomatic sublingual acinar tissue if tunneling dorsal to the digastricus muscle and dissection to the sublingual caruncle is not performed during mandibular‐sublingual sialadenectomy in cats. We hypothesized that without tunneling and rostral dissection the presence of residual polystomatic salivary acinar tissue would be confirmed histologically in most (> 50%) cases.

## 2 MATERIALS AND METHODS

### 2.1 Specimens

A total of 10 feline cadavers euthanized for reasons unrelated to this study and donated from a local shelter were included. Sex, breed, and postmortem bodyweight were recorded. Ethical approval was obtained from the primary author's institution (AREC‐E‐20‐16‐Mullins). Cadavers were stored in a −20°C freezer until thawed for use. Then, 24 h prior to surgery, cadavers were defrosted at room temperature.

### 2.2 Randomization of approach, surgical technique, and histologic analysis

For the first cat to be operated, a coin was tossed to determine whether the left side would be operated by a ventral or ventrolateral approach. The other approach was performed on the contralateral side. The approach was alternated between left and right side for each of the remaining nine cats.

**FIGURE 1:**
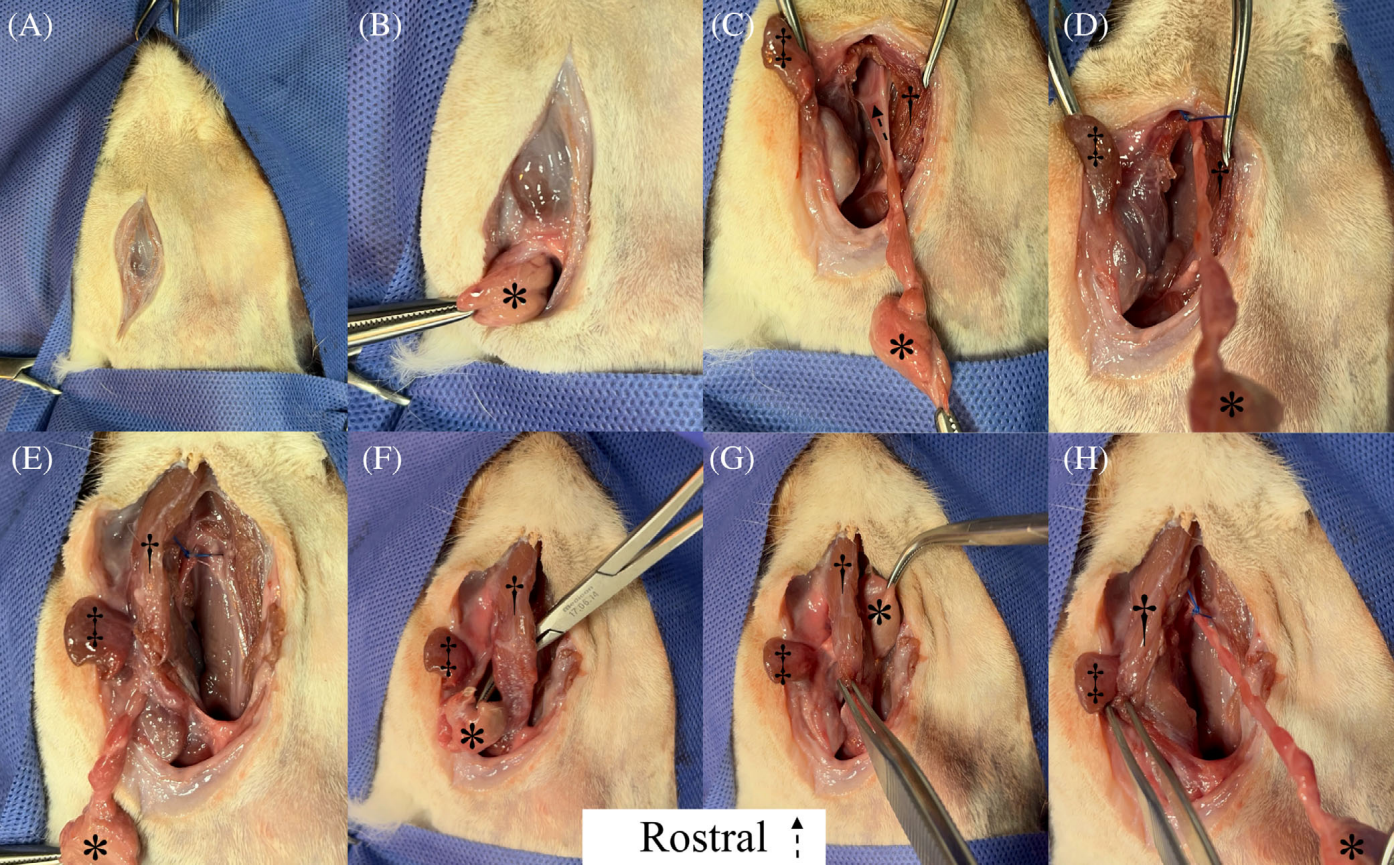
Intraoperative images of a mandibular‐sublingual sialadenectomy via the ventral approach. (A) Surgical incision caudal to the angular process of the mandible toward the mandibular symphysis. (B) Following blunt dissection, the mandibular salivary gland (asterisk) is identified and grasped with a hemostatic forceps. Ductal tissue is dissected as far rostral as possible while the mandibular‐sublingual salivary gland‐duct complex is pulled caudally. (C) Note the position of the ipsilateral lingual nerve (arrow) traversing the salivary ducts and mandibular lymph node (double dagger). (D) At the point of maximal exposure of the salivary ducts without tunneling, a ligature is placed. (E) Blunt dissection is performed medial and dorsal to the digastricus muscle (dagger). (F) A hemostatic forceps is passed from medial to lateral dorsal to the muscle, exiting alongside the mandibular salivary gland. (G) The mandibular salivary gland is pulled from lateral to medial dorsal to the digastricus muscle. (H) Rostral dissection is performed toward the sublingual caruncle. Rostral is to the top of the image.

**FIGURE 2:**
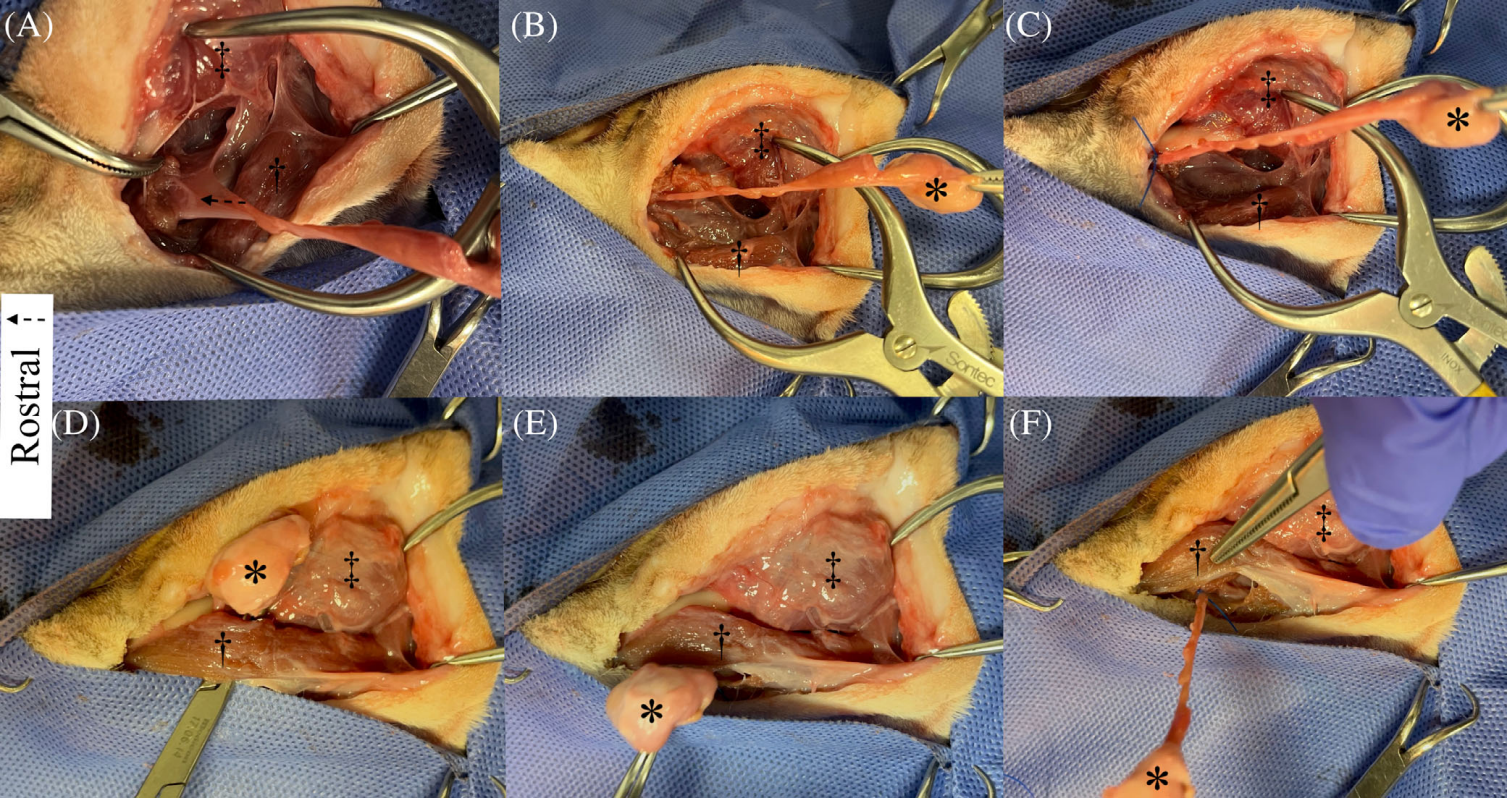
Intraoperative images of a mandibular‐sublingual sialadenectomy via the lateral approach. (A) Dissection of mandibular‐sublingual salivary gland‐duct complex after skin incision. Note the position of the ipsilateral lingual nerve (arrow) traversing the salivary ductal tissue (indicated by tips of hemostatic forceps). (B) Salivary ducts are dissected as far rostral as possible until they travel dorsal to the digastricus muscle (dagger). (C) At the point of maximal exposure of the salivary ducts without tunneling, a ligature is placed. (D) After blunt dissection medial and dorsal to the digastricus muscle, a hemostatic forceps is passed from ventral to dorsal under the muscle, exiting alongside the mandibular salivary gland. Note the attachment of the digastricus muscle to the ventral aspect of the mandible and the position of the masseter muscle (double dagger). (E) The mandibular salivary gland is pulled from dorsal to ventral under the digastricus muscle. (F) Dissection is continued in a rostral direction toward the sublingual caruncle. Rostral is to the left of the image.

**FIGURE 3:**
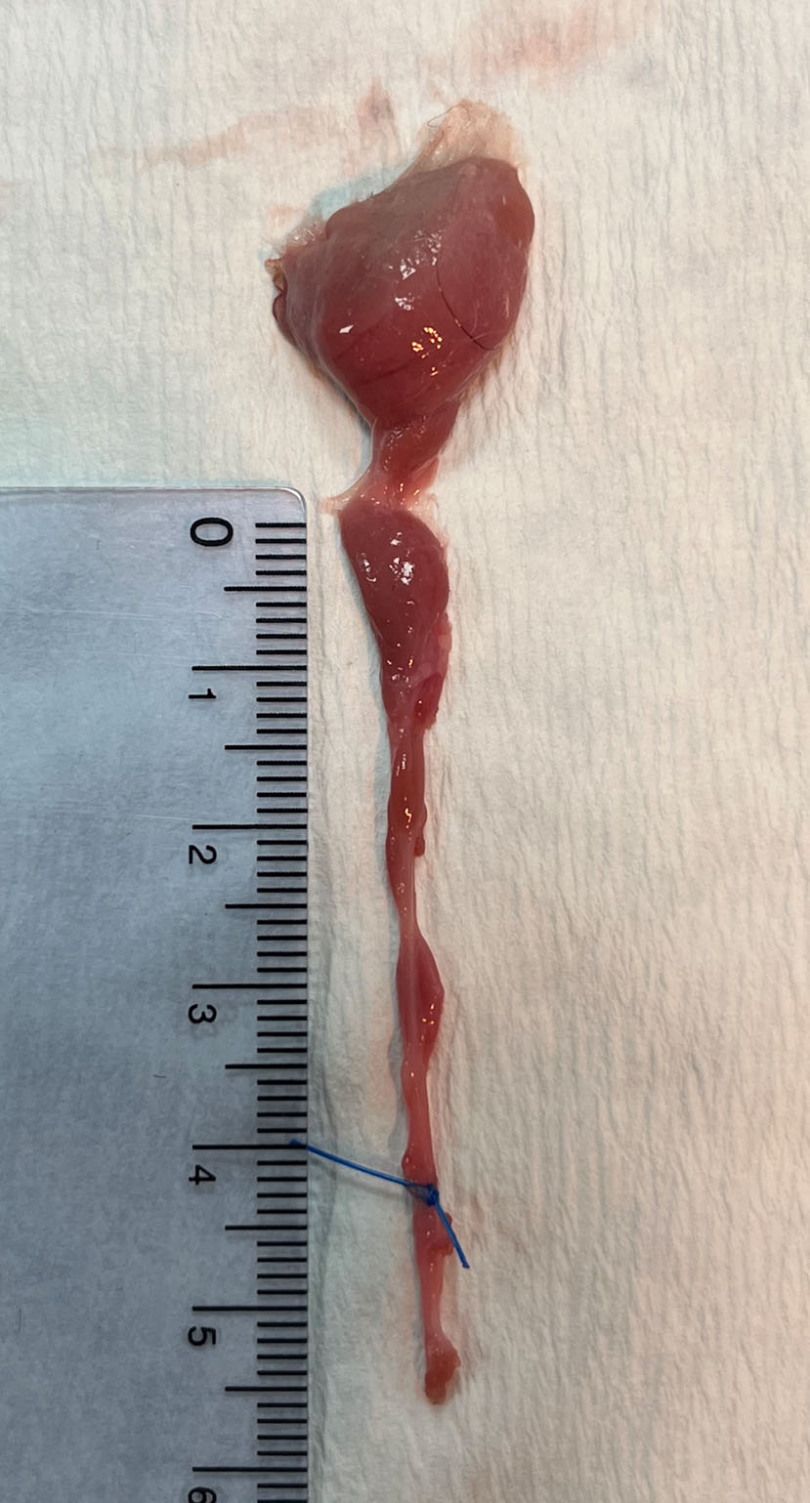
Excised mandibular and sublingual salivary gland‐duct complex.

All surgeries were performed by a board‐certified small animal surgeon (RAM). For the ventral approach, cats were positioned in dorsal recumbency, whereas for the ventrolateral approach, cats were positioned in oblique lateral recumbency with the side to be operated uppermost. For the ventral approach, a straight skin incision was made from caudal to the angular process of the mandible to just caudal to the mandibular symphysis.^5^, ^6^ For the ventrolateral approach, a curvilinear skin incision was made just caudal to the angular process of the mandible and later extended rostrally toward the mandibular symphysis.^7^ Subcutaneous tissue and platysma were incised along the same line. Dissection was performed caudal to the mandibular ramus until the mandibular salivary gland was identified. On opening the capsule, the gland was grasped with a hemostatic forceps and salivary tissue dissected as far rostral as possible, with retraction of the digastricus and masseter muscles. Where the ducts traveled dorsal to the digastricus muscle and no further duct tissue could be exposed, a ligature was placed around the ducts (Figures 1 and 2). Blunt dissection was performed medial and dorsal to the digastricus muscle and a hemostatic forceps was passed from medial to lateral (ventral approach) or ventral to dorsal (ventrolateral approach) dorsal to the muscle, exiting alongside the mandibular salivary gland. The mandibular salivary gland was pulled dorsal to the digastricus muscle and dissection continued in a rostral direction, rostral and dorsal to the lingual nerve. The mylohyoideus muscle was incised and the salivary ducts dissected to the level of the sublingual oral mucosa where they were transected with Metzenbaum tissue scissors (Figures 1 and 2). The MS‐GDCx was laid out straight on a sheet of paper (Figure 3) and the length of dissected tissue rostral to the ligature was carefully excised and placed in 10% neutral‐buffered formalin. Following fixation, each tissue specimen was processed and embedded in paraffin, sectioned at 4 μm, and stained with hematoxylin and eosin. Histologic sections were assessed for the presence of salivary acinar tissue by a board‐certified veterinary pathologist (PAK).

### 2.3 Statistical analysis

Continuous data were tested for normality using the Shapiro–Wilk test. Normally distributed continuous data are presented as mean and SD and were compared using the paired *t*‐test. Categorical data are presented as frequencies and percentages. The rate of residual polystomatic sublingual acinar tissue was defined as the percentage of specimens in which salivary acinar tissue was identified on histologic examination and was compared between approaches using the Fisher's exact test. A *p‐*value less than .05 was considered statistically significant. Statistical analyses were performed using GraphPad (https://www.graphpad.com/).

## 3 RESULTS

A total of 10 adult feline cadavers were included in the study. There were seven males and three females. All 10 cadavers were domestic shorthairs. Mean (SD) postmortem bodyweight was 3.6 (0.7) kg.

The mean (SD) length of tissue rostral to the ligature (Figure 3) was 12.5 (3.4) mm with the ventral approach and 10.7 (6.4) mm with the ventrolateral approach. In all cases, the lingual nerve could be identified in the region of where the ligature was placed at the most rostral extent of the dissection with the digastricus muscle retracted without tunneling.

**FIGURE 4:**
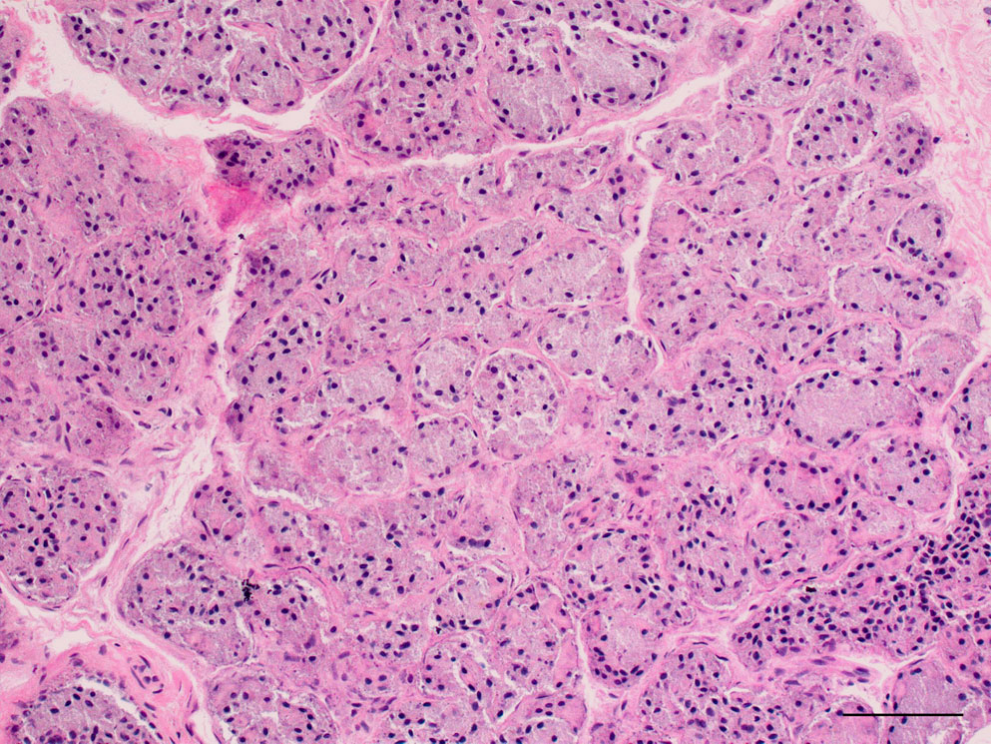
Lobules of salivary gland with numerous acini located along a salivary duct. Size bar = 25 μm.

Histologic assessment of 19 tissue specimens was performed. The specimen from the right side of cat 9 (ventrolateral approach) was misplaced between surgery and processing, and histology could not be performed. Overall, histology identified evidence of residual polystomatic sublingual acinar tissue in 13 (68.4%) of 19 specimens, and the presence of ductal tissue alone in the remaining six (31.6%). For the ventral approach, histology identified evidence of residual polystomatic sublingual acinar tissue in seven (70.0%) of 10 (Figure 4) specimens, and the presence of ductal tissue alone in the remaining three (30.0%). For the ventrolateral approach, histology identified evidence of sublingual acinar tissue in six (66.7%) of nine specimens, and the presence of ductal tissue alone in three (33.3%). There was no significance difference in the rate of residual sublingual acinar tissue between ventral and ventrolateral approaches (*p* > .99).

## 4 DISCUSSION

The main finding of this study is that the presence of residual polystomatic sublingual acinar tissue rostral to the point of tunneling was confirmed histologically in the majority (68.4%) of cases, 70.0% with the ventral approach and 66.7% with the ventrolateral approach. On the basis of these findings, we accept our study hypothesis.

The findings of our study are in agreement with those of a previous canine cadaveric study^16^ in which investigators compared the presence of residual polystomatic sublingual salivary tissue before and after tunneling using a lateral approach. Similar to our study, tunneling was found to be associated with a significant reduction in the presence of residual gross lobules of salivary tissue.^16^ In that study,^16^ excision was performed as far rostral as possible after tunneling but not necessarily to the sublingual caruncle, which resulted in two dogs having persistence of a small amount of salivary tissue rostral to the level of where resection was performed. This highlights that tunneling does not eliminate the possibility of residual salivary tissue if dissection does not extend sufficiently rostral. In a retrospective study^17^ that compared the rate of sialocele recurrence after ventral versus lateral sialadenectomy in dogs, 5/70 dogs in the lateral group experienced recurrence, four of which had had tunneling performed, compared with 0/70 dogs in the ventral group. In both groups, salivary gland‐duct complexes had been ligated and transected as close as possible to the lingual nerve at initial surgery. Incomplete removal of the most rostral salivary tissue was confirmed at revision surgery in all cases.^17^ After tunneling was performed in our study, dissection continued to the sublingual oral mucosa and therefore we did not obtain further tissue samples to determine if there was histologic evidence of salivary acinar tissue more rostrally.

Surgical excision of the affected salivary gland is the recommended treatment in most cats with sialoceles.^1^, ^2^ In two of the largest studies to date,^1^, ^2^ sialoadenectomy of the affected salivary gland was performed in 57.9%–81.0% of cases. Marsupialization of sublingual sialoceles (ranulas) has also been described, either alone or in combination with sialadenectomy.^2^, ^5^, ^6^, ^12^ To the authors' knowledge, there are no reports of sialocele recurrence following sialadenectomy or marsupialization in the feline literature, with variable follow‐up ranging from 0.5 months to 13 years.^1^, ^2^, ^3^, ^5^, ^12^ Tunneling dorsal to the digastricus muscle has previously been described in clinical cases of sialoceles in cats, with both a ventral and lateral approach.^1^ In one study,^1^ investigators used the tunneling technique to permit salivary duct ligation as close as possible to the lingual nerve. This nerve has been described as the junction of the monostomatic and polystomatic parts of the sublingual salivary gland.^14^ In a primary surgical textbook,^14^ it is recommended that dissection should continue rostral to the lingual nerve dorsal to the mylohyoideus if a ranula exists to remove all glandular tissue up to the sublingual caruncle. In our study, the lingual nerve could be identified at the most rostral extent of the dissection without tunneling. Other investigators have described techniques such as applying rostral traction on the digastricus muscle or tractioning of the gland‐duct complex with dissection dorsal to the digastricus muscle to achieve rostral dissection without tunneling.^5^, ^6^ These reports have limitations including lack of follow‐up,^6^ lack of information regarding the length of salivary duct excised or the location of the lingual nerve,^6^ and lack of information regarding the exact level salivary gland‐duct tissue was transected.^5^

In a previous study^16^ involving a lateral approach in canine cadavers, tunneling dorsal to the digastricus muscle was associated with a median increase in length of salivary duct exposure of 1.8 cm. In our study, tunneling dorsal to the digastricus muscle was associated with a 10.7–12.5‐mm mean additional length of salivary duct exposure, however, the length of the duct has much less relevance that the presence of acinar tissue. An important limitation we acknowledge is the uncertainty regarding whether any sublingual salivary acinar tissue left in situ will lead to sialocele recurrence in clinical cases. However, the ultimate goal of a sialoadenectomy is to remove all glandular tissue to minimize the risk of recurrence, particularly in cases presenting with a ranula. Therefore, we believe that by tunneling dorsal to the digastricus muscle and continuing dissection further rostrally, a more complete excision of both ductal and acinar tissue can be achieved, thereby potentially reducing the likelihood of recurrence in clinical cases. This assumption is supported by our histologic findings, as acinar tissue was identified in the majority of tissue specimens submitted for evaluation. These specimens represent tissue that would have remained in situ had tunneling not been performed, suggesting that this approach may play an important role in achieving a more complete glandular removal.

Similar to a previous study in dogs,^16^ retraction of the masseter and digastricus muscles was performed in our study to maximize exposure of salivary ducts and allow fair assessment of the rate of residual acinar tissue without tunneling. It is likely that retraction of these muscles would be easier in cats than in dogs on the basis of their smaller size. In contrast to the study by Marsh and Adin,^16^ we placed a ligature instead of injecting ink at the level of where salivary ducts traveled dorsal to the digastricus muscle because of concerns that the ink would become more diffuse with tunneling and result in less accurate measurements.

We recognize several important limitations of our study. Some of the specimens were affected by autolysis; however, histologic assessment for acinar tissue was still possible. Furthermore, it is possible that the cadaveric nature of our study may have altered the tissue properties and increased the exposure that could be achieved without tunneling. Freezing and defrosting associated with the cadaveric nature of the study may have changed the consistency of the tissues and made dissection more difficult compared to a clinical case. The ligature in our study was placed at the point of maximal duct exposure without tunneling; however, because this point is not based on specific anatomic landmarks, it is possible that different results would be obtained if the study were to be repeated. However, all of the surgeries were performed by a single board‐certified surgeon, which should offer consistency in terms of how much the tissues were retracted and where the ligature was placed. The effect of tunneling on the rate of intra‐ and postoperative complications and the clinical benefit of performing this technique cannot be assessed. All of the cats included in our study were domestic shorthair and therefore our results may not be representative of other breeds.

Histology identified evidence of residual polystomatic sublingual acinar tissue in the majority of cases in our study, with no difference between ventral and ventrolateral approaches. This would suggest that tunneling dorsal to the digastricus muscle and excision as far rostral as possible would result in a lower rate of sublingual salivary acinar tissue being left in situ after sialadenectomy in cats with sialoceles related to the MS‐GDCx. Further clinical studies, likely multi‐institutional on the basis of the uncommon nature of this condition in cats and with appropriate patient follow‐up, are required to investigate whether tunneling actually translates into a lower rate of sialocele recurrence in vivo.

## AUTHOR CONTRIBUTIONS

Mullins RA, MVB, DVMS, DECVS, PGDipUTL: Designed the study; led the data collection and analysis; performed all statistical analyses; wrote the first draft of the manuscript; read, revised, and approved the final version of the manuscript. Marirrodriga Larrocha I, DVM, Ortega Jusdado C, DVM, PgCertSAS and Otero Balda I, DVM, MSc: Contributed to data collection; read, revised, and approved the final version of the manuscript. Kelly PA, BSc, MVB, PhD, FRCPath, DECVP, PGDipUTL: Performed histologic analysis; read, revised, and approved the final version of the manuscript.

## FUNDING INFORMATION

No funding was received for this study.

## CONFLICT OF INTEREST STATEMENT

The authors have no conflict of interest to declare.
